# Bisphosphonates for osteoporosis: from bench to clinic

**DOI:** 10.1172/JCI179942

**Published:** 2024-03-15

**Authors:** Teresita Bellido

**Affiliations:** Department of Physiology and Cell Biology and Winthrop P. Rockefeller Cancer Institute, University of Arkansas for Medical Sciences, Little Rock, Arkansas, USA. Central Arkansas Veterans Healthcare System, John L. McClellan Little Rock, Little Rock, Arkansas, USA.

In honor of the 100th anniversary of the *Journal of Clinical Investigation* (*JCI*), I am pleased to provide this Viewpoint on bisphosphonates, which are, without doubt, one of the most powerful drugs used in the treatment of bone diseases.

Bisphosphonates’ high affinity for bone mineral hydroxyapatite makes them tissue specific and inherently devoid of off-target effects on other tissues. Bisphosphonates are analogs of inorganic pyrophosphate (P), in which the oxygen (O) of the P-O-P chemical structure is replaced by a carbon (C), leading to the P-C-P structure. This change provides the opportunity of adding two chemical groups to the C, leading to the synthesis of alternative analogs with different potencies and varying affinities for the bone mineral.

## From pipe cleaners to powerful bone-protective agents

The history of bisphosphonates is fascinating, full of surprises, and an example of how simple basic science facts can advance clinical medicine and profoundly affect population health.

Taking advantage of their properties to chelate calcium and inhibit calcium carbonate precipitation, the drugs were initially used in the early 1970s as anticorrosive agents to remove calcium scales from industrial pipes (1). Their potential dental and medical applications were recognized soon after the discovery of their ability to inhibit not only the formation, but also the dissolution, of hydroxyapatite crystals and to hinder bone resorption (2).

The first-generation bisphosphonates (etidronate and clodronate) were introduced in the clinic in the 1970s and 1980s, and the later generation the bisphosphonate alendronate was approved by the FDA in 1995. Today, several bisphosphonates are the first-line treatment to stop bone loss in diseases presenting with exaggerated bone resorption, including all forms of osteoporosis, Paget’s disease of bone, aging, and cancer in bone.

## Biological targets of bisphosphonates

In the early 1990s, when it was clear that bisphosphonates’ physicochemical properties were not sufficient to explain their mechanism(s) of action, there was an explosion of biological in vitro, in vivo preclinical, and clinical research that provided the mechanistic basis for their bone-protective activity.

These studies led to the identification of osteoclasts, the bone-resorbing cells, as targets of bisphosphonate action, and the enzyme farnesyl pyrophosphate synthase (FPPS) of the mevalonate pathway was shown to be a major molecular target of the drugs. Inhibition of this pathway by bisphosphonates leads to osteoclast detachment from the bone surface and termination of bone resorption (1), resulting in preservation of skeletal mineral and maintenance of bone mass.

## Quantity versus quality: promoting bone strength by maintaining osteocytes

With increased usage, it was soon evident that the decrease in bone fracture incidence induced by bisphosphonates was disproportional to their antiresorptive properties and effects on bone mass, suggesting an additional effect on bone strength unrelated to the drugs’ actions on osteoclasts.

During this time of increased use, in the late 1990s, another bone cell was coming to the center stage: the osteocyte (3, 4). Osteocytes — the most abundant bone cells — were hypothesized to detect damaged bone and orchestrate its removal through the sophisticated osteocytic network, expanding the entire mineralized bone matrix and reaching to the bone surfaces. However, how osteocytes buried within the mineral could coordinate bone repair was not understood. With the generation of new osteocytic cell lines and the development of unique molecular means to target osteocytes in animal models, an avalanche of research demonstrated that untimely death of osteocytes could account for disruption of this network, leading to decreased bone quality and increased bone fragility. It was also shown that accumulations of apoptotic osteocytes mark areas of bone that need to be replaced, signal to osteoclast precursors, and initiate “targeted” remodeling, i.e., bone resorption in particular areas of the skeleton that need replacement (5).

**Figure 1:**
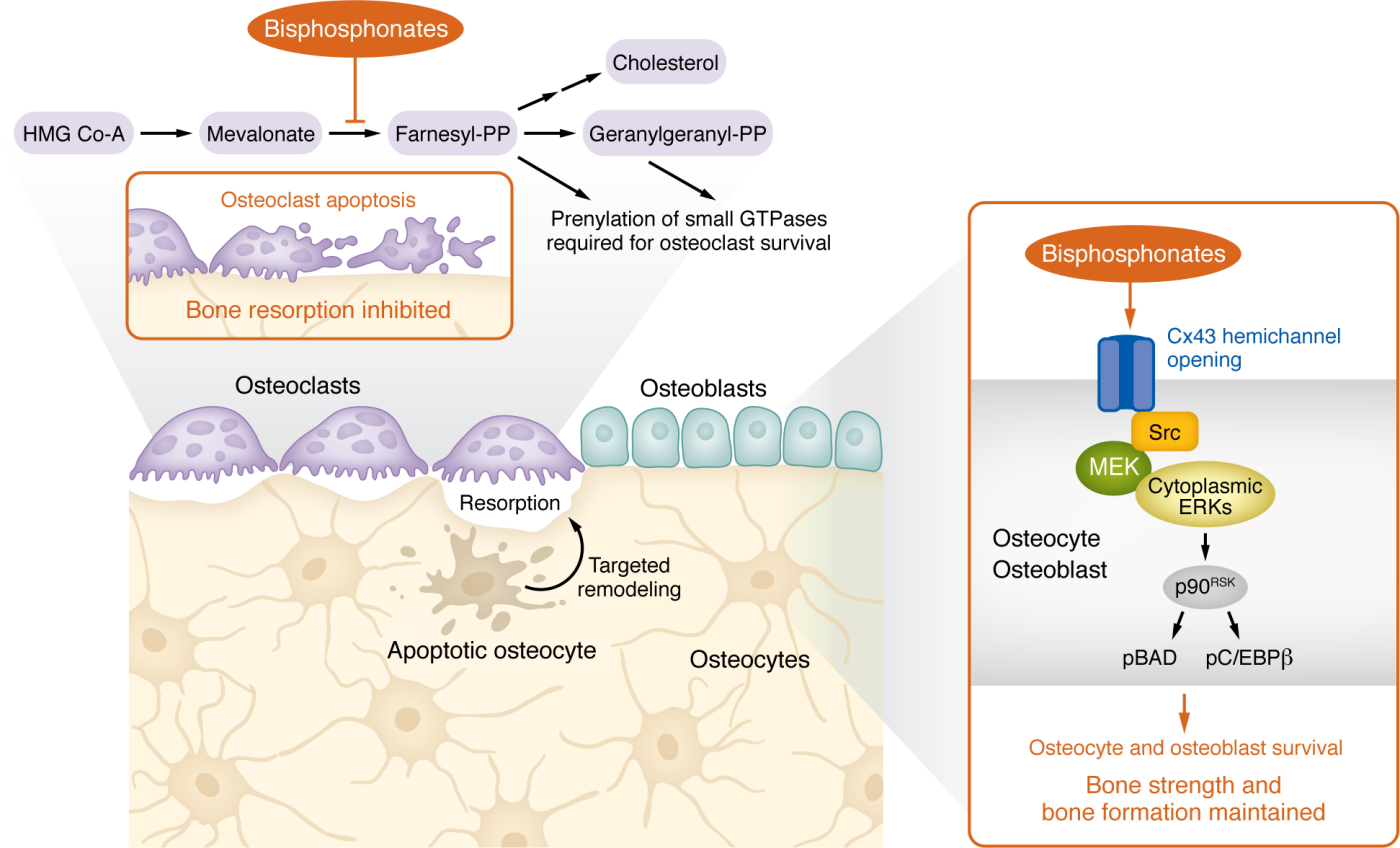
Distinct biological actions of bisphosphonates on bone cells explain the protective effects of the drugs on the skeleton. Bisphosphonates inhibit the mevalonate pathway in osteoclasts, inducing their apoptosis, leading to inhibition of bone resorption. Additionally, the drugs open Cx43 hemichannels, promoting survival of osteocytes and osteoblasts, maintaining bone strength and bone formation.

In 1999, work from my lab published in the *JCI* showed that osteocytes (and osteoblasts) were target cells of bisphosphonates and that the drugs prevented the increased prevalence of apoptosis of these cells induced by excess of glucocorticoids (6) (Figure 1).

This paper had an unanticipated impact in the field because it dismantled a few then widely accepted notions. Furthermore, the research that followed, by our laboratory and the research community in general, changed forever the perception of bisphosphonates as monodimensional drugs. These findings continue to reverberate today. Personally, as a junior faculty at that time, I learned to trust my instincts, follow the data, and interpret research findings with candor and without fear. The work was also a demonstration of team science and provided important lessons on how to collaborate effectively, lessons that I have embraced in my scientific career.

Our work was simple and at the same time remarkable. It provided irrefutable evidence that osteoclasts were not the only bone target cells of bisphosphonates and that osteocytes contribute to bone strength by mechanisms beyond the control of bone mass. Furthermore, it was clear that, besides the recognized direct effect of the drugs on osteoclasts, bisphosphonates interfere with remodeling indirectly by preserving osteocyte viability and thus regulating targeted remodeling.

Another startling outcome of our research was the recognition that the molecular mechanism of the antiapoptotic effect on osteocytes/osteoblasts was unrelated to interference with the mevalonate pathway and that the antiapoptotic effect on osteocytes and osteoblasts was exerted at much lower concentrations than those needed for the effect of bisphosphonates on osteoclasts (6). This discovery opened the possibility that the integrity of the osteocyte network could be maintained without affecting osteoclasts directly, thereby avoiding an excessive decrease in remodeling. Indeed, through biological screens, we discovered osteocyte/osteoblast-selective bisphosphonate analogs that preserve the osteocyte network, bone formation, and bone strength without decreasing bone resorption (7).

## Connexin-43 and hemichannels: novel biological targets of bisphosphonates

Our work also identified the gap junction protein connexin-43 as required for bisphosphonates’ antiapoptotic effects on osteocytes/osteoblasts (8). Surprisingly, survival induced by bisphosphonates does not require cell-to-cell interactions but instead is mediated by opening of connexin-43 hemichannels, half-gap junction channels hitherto considered nonfunctional in bone cells (or any other cell types) (9, 10). Hemichannel opening by bisphosphonates triggers a novel cell survival pathway driven by connexin-43 through its interaction with the kinases Src and ERKs. This finding explained the exclusive requirement of connexin-43 for the effect of bisphosphonates, as no other member of the connexin family possesses the ability to interact with Src and thus activate the ERK pathway.

Our discovery of a connexin-43 hemichannel/Src/ERK pathway opened lines of research that culminated in the finding that mechanical signals are the endogenous cues that open connexin-43 hemichannels under physiological conditions leading to osteocyte/osteoblast survival (11). Thus, another consequence of the research on the mechanism of action of bisphosphonates is learning about the role of connexin-43 hemichannels in bone mechanotransduction.

## The good and the bad

After several years of bisphosphonate use, it was clear that the potent antiresorptive effects of bisphosphonates had some undesired effects. Long-term use of the drugs in patients was associated with rare cases of osteonecrosis of the jaw and atypical femoral fractures. The evidence that treatment with another strong inhibitor of resorption, the antibody neutralizing RANKL, producing similar side effects supports the notion that potent inhibition of bone remodeling underlies these unwelcome effects.

This evidence prompted the field to revise the recommendations for bisphosphonate treatment, decreasing the duration of the treatment and/or implementing “drug holidays” in particular in patients with lower risk of fracture (12). Nevertheless, the benefits outweigh the risks, and if patients are at high risk of suffering a bone fracture, receiving bisphosphonates is more likely to prevent a fracture than to induce the adverse side effects (13).

Another “good” coming from the “bad” of bisphosphonate action(s) is the identification of mutations in enzymes of the mevalonate pathway that increase the risk of atypical femoral fractures, which provided an opportunity to tailor antiresorptive treatments to patients’ genetic/epigenetic profiles (14).

## Bisphosphonate-bone specificity: from diagnostic to bone-targeting tools

The bone-seeking properties of bisphosphonates have been advantageously employed as diagnostic tools using radiolabeled bisphosphonates to image sites of active bone remodeling with positron emission tomography (PET) and to trace sites of bone metastasis using single-photon emission computed tomography (SPECT) (15).

Another use of bisphosphonates developed in recent years is based on their ability to direct other drugs specifically to the bone microenvironment (16). Bisphosphonate analogs maintaining their affinity for the bone mineral but devoid of (or with low) antiresorptive activity have been conjugated to drugs targeting specific signaling pathways in bone by means of pH-sensitive linkers that assure their delivery to active bone surfaces. This maneuver increases the therapeutic efficacy of the drug in question and circumvents adverse effects on other tissues.

Examples of this “target-and-release” mechanism are the successful delivery of inhibitors of the proteasome that increase their bone anabolic effectiveness (17) as well as inhibitors of the Notch pathway that correct the bone disease induced by multiple myeloma (18) and increase the bone gain induced by parathyroid hormone (19). Furthermore, conjugation of bisphosphonates to antibiotics has proved to exert antimicrobial activity and effectively treat osteomyelitis in preclinical models (20). A remarkable finding is that the antibiotics can not only kill microbes on the bone/bone marrow areas, but also those present in the osteocyte lacunae and canaliculi, indicating the ability of the conjugates to deliver drugs to the osteocytic network.

## Implications and impact of the research on bisphosphonates

The implications of the research on bisphosphonates are indisputable. Bisphosphonates are an example of how basic science research can be effectively leveraged to improve the lives of patients. The discovery of the bone-seeking properties and the bone-protective effects of bisphosphonates that took place more than 40 years ago continues to have a major impact in the bone field today. These drugs are one of the primary therapeutic and diagnostic tools for bone diseases. And the future is bright, as novel applications, like targeting drugs specifically to bone, are just starting to emerge and promise to effectively treat bone infections and restore bone health in patients with cancer. There is little doubt that research on the mechanisms of action of bisphosphonates will continue to add to our current knowledge of the molecular and cellular biology of bone and how to treat and prevent human diseases.

## Version 1 03/15/2024

Electronic publication
